# Effectiveness of universal secondary school-based interventions addressing the psychological risk factors of eating disorders in adolescents: a systematic review

**DOI:** 10.1186/s40337-026-01712-9

**Published:** 2026-08-21

**Authors:** Melissa Stock, Robyn Murphy, Joanna Adams

**Affiliations:** 1 Oxford Institute of Clinical Psychology Training and Research, Medical Sciences Division, University of Oxford, Oxford, UK; 2 Oxford Health NHS Foundation Trust, Oxford, United Kingdom

**Keywords:** Eating disorders, Adolescents, School-based interventions, Early intervention, Universal prevention, Systematic review

## Abstract

**Objective:**

To evaluate the effectiveness of universal secondary-school interventions targeting core eating disorder (ED) psychopathology and at least one additional psychological risk factor.

**Method:**

A systematic search of databases was conducted, including Scopus, CINAHL, Embase, PsycINFO, MEDLINE and Google Scholar in March 2025. Eligible randomised controlled trials (RCTs) reported quantitative outcomes (e.g., ED psychopathology, body dissatisfaction, dieting behaviors) following universal secondary-school interventions addressing weight, shape, or eating concerns and another psychological risk factor in non-clinical adolescents aged 11–18. Results were synthesized narratively, and study quality was evaluated using the Cochrane Risk of Bias (RoB-2).

**Results:**

Seventeen RCTs were included. Eleven studies were conducted in Europe, most participants were female and in middle adolescence. Overall, most universal school-based ED interventions produced some immediate improvement in ED symptoms or well-being but effects were generally small or rarely sustained at follow-up. There were no significant changes in BMI. However, the high risk of bias limits the confidence of findings.

**Discussion:**

These improvements were short term, and their effects were mostly small. Future research would benefit from adopting active comparison groups and longer follow-ups. Whilst core ED psychopathology in addition to the risk factors of body image dissatisfaction, dieting, self-esteem, and social media appear consistent factors to include in universal interventions, further understanding of the most effective risk factors to target in this population is needed.

## Abstract (plain language summary)

Adolescence is a time of significant change, and this period often coincides with the greatest increase in symptoms of disordered eating and eating disorders. Universal programs are commonly used in schools to help with early identification and prevention of such difficulties. This systematic review explores the effectiveness of universal, school-based interventions that target core symptoms and risk factors associated with eating disorders in adolescent populations. Seventeen research papers were included in the review. Overall, the findings show mixed results on how well the interventions reduce eating disorder symptoms and improve general well-being, with no changes found in BMI. We need to better understand which risk factors are most important to focus on for helping young people.

## Introduction

Disordered eating behaviors, including dieting, use of compensatory behaviors (e.g., self-induced vomiting or excessive exercise) are increasing rapidly in adolescents [61]. With adolescence being a developmentally sensitive time [72], around 12.3% of 11–16-year-olds in the United Kingdom (UK) screened positive for eating difficulties in 2023, up from 6.7% in 2017 [83]. More globally, 1 in 5 children and young people (CYP) displayed disordered eating across 16 countries, with behaviors significantly higher in cisgender girls than boys [61]. Furthermore, disordered eating is rising in several minoritised groups including those from lower-income backgrounds, transgender and gender diverse, and ethnic groups [44, 58, 90, 97].

While disordered eating can vary in severity, behaviors do not always meet criteria for an eating disorder (ED) diagnosis [26, 108]. However, such behaviors can be significant predictors for the onset of EDs [12, 67]. Disordered eating and EDs can cause significant psychological and physical impairments [10, 120], with mortality rates in EDs higher than any other mental health (MH) condition [9].

Whilst there are CYP treatment pathways, significant environmental, individual, and systemic barriers to accessing services remain [46]. Remission rates remain low [73] and low help-seeking behavior and high rates of drop out from treatment result in prolonged illness and increased healthcare costs [8, 27]. Yet emerging research suggests preventative interventions for CYP, targeting the transdiagnostic core psychopathology of EDs and its risk factors, can improve dietary restraint, thin-ideal internalization, body dissatisfaction, and disordered eating [17, 88, 95, 101]. Taken together, this suggests that future research would benefit from focusing on effective and practical strategies for prevention and early intervention [56].

Prevention programs aim to minimise psychopathology and associated risk factors of health conditions and are classified as targeting either a ‘universal’, ‘selective’, or ‘indicated’ population [119]. Universal programs target a whole population group that have not been identified as being at risk; selective programs target individuals at risk of developing a condition; whilst indicated programs are brief, targeted interventions provided to individuals showing early signs of difficulty but not meeting criteria for full treatment [119]. For CYP, schools are considered appropriate settings for prevention programs [3], thus clinicians and policymakers worldwide advocate for schools to incorporate universal interventions to promote good mental and physical health [12,21, 78, 86].

Grounded in extensive empirical and theoretical work, including the biopsychosocial model [23], EDs are understood to arise from a combination of biological, psychological, and social risk factors. Biological contributors include gender, developmental stage, ethnicity, low Body Mass Index (BMI), genetic vulnerability, and family history of EDs [10, 100, 113]. Social factors such as weight stigma, loneliness, and bullying also play a significant role [10, 98]. Psychological risk factors encompass a range of modifiable processes, including body image concerns, perfectionism, negative affect, low self-esteem, comorbid conditions (e.g., anxiety/low mood), social media, and dieting or exercise-related behaviors [10, 98, 100], with the transdiagnostic core processes of eating, weight, and shape concern recognised as particularly central [25]. These core processes are consistently identified as among the strongest contributors to the development and maintenance of disordered eating and EDs [12, 25, 74], highlighting their importance as key targets for early prevention, particularly within educational settings.

Recognising the central role of these modifiable psychological risk factors provides the foundation for considering how existing school‑based interventions address them. Previous reviews highlight the need for theory-driven interventions [3, 56, 82]. Two systematic reviews have evaluated the effectiveness of school-based body image interventions, suggesting improvements in body image and esteem [17, 121]. Interventions were more effective when delivered separately for boys and girls [121], with girls benefitting more than boys [17]. However, drawing conclusions is difficult as effect sizes were small, and quality of evidence low. In another review, Berry and colleagues [11] found, when focusing solely on ED symptomatology, secondary school-based interventions did not reliably demonstrate effectiveness compared to control groups over time. In contrast, Pursey et al.’s [88] multi-risk factor primary-school ED prevention review revealed improvements in body image-related outcomes, with some progress in weight control and eating behaviors. Thus, Berry and colleagues [11] suggest broadening the scope of school-based interventions to address both core ED psychopathology and risk factors.

Therefore, this current review aims to evaluate the effectiveness of universal secondary school-based interventions that address the transdiagnostic core ED psychopathology of eating, weight, or shape concern, in addition to at least one other modifiable psychological risk factor including body image concerns, perfectionism, negative affect, low self‑esteem, comorbid conditions, and dieting or exercise‑related behaviours. These psychological risk factors were included because they consistently emerge as common, modifiable contributors to ED risk across the rapid review literature. The primary outcome measure of interest was self-reported ED psychological outcomes (e.g., Eating Disorder Examination Question [EDE-Q]) [24], which assesses core ED psychopathology, including cognitions and affect related to eating, weight, and shape concerns, as well as associated behaviours such as restraint and at least one psychological risk factor. We were also interested in the effect interventions have on physical health outcomes (e.g., BMI) and broader psychological well-being outcomes (e.g., depression and anxiety).

## Method

### Protocol and registration

This review was pre-registered with PROSPERO (CRD42024517930) and performed according to Preferred Reporting Items for Systematic Reviews and Meta-Analyses (PRISMA) guidelines [85]. See Supplementary Materials 1 for PRISMA checklist. Inclusion criteria and quality assessment were amended during the review process and recorded appropriately.

### Eligibility criteria

Inclusion criteria is displayed in Table 1. Non-English studies and gray literature were excluded due to resource constraints and to prioritise peer-reviewed studies with rigorous methodological standards and verified reliability.

### Search strategy

Search terms (See Supplementary Materials 2) were co-developed with a librarian and by reviewing Pursey et al.’s [88] search terms. Key terms included: eating disorder*, body image*, psychosocial factor*, intervention*, adolesc*. Searches were conducted across databases (Scopus, CINAHL, Embase, PsycINFO, MEDLINE) and Google Scholar by the first author (MS) in September 2024 and March 2025. Alerts were created (closed 22/05/2025) to identify newly published articles. Reference lists were searched and researchers were contacted for any missed studies.

### Selection process

**Fig. 1:**
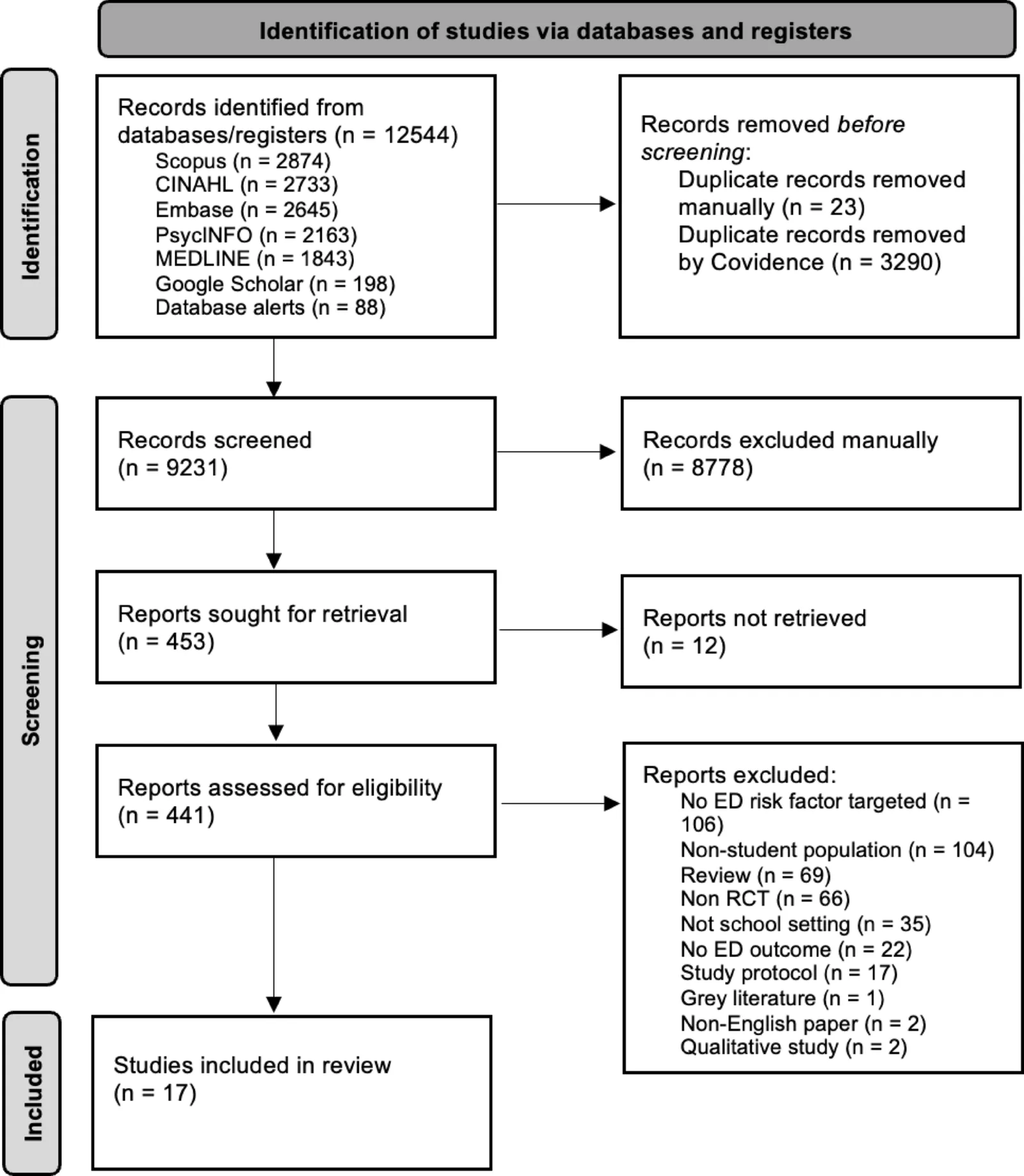
PRISMA Flow Chart created using Haddaway et al. [45] online tool

A total of 12,456 titles and abstracts were retrieved from the initial search (Fig. 1). Duplicates (*n* = 3,313) were removed manually and using Covidence [19]. A second reviewer (RM) screened 20%, with inter-rater reliability κ = 0.826 [70]. Before full text screening, study design criteria was refined to include only RCTs to improve methodological rigour of the review and maintain feasibility given the unexpected high number of eligible studies. MS screened selected full texts (*n* = 453) with RM screening 20%. Inter-rater reliability was κ = 0.849 [70]. Twelve papers were irretrievable despite author’s efforts in contacting researchers and university/National Health Service (NHS) libraries. Discrepancies were discussed between authors. Eighty-eight additional papers were identified from database alerts and updated searches, with titles and abstracts screened separately. No additional papers met criteria.

### Data extraction

A data extraction form was developed and tested to ensure all relevant information was charted. The remaining articles (*n* = 17) were included for data extraction and information comprised (a) study location and language; (b) design and analysis; (c) intervention and setting; (d) sample characteristics; (e) core psychopathology and risk factor measures; (f) outcomes. Researchers were contacted to gather absent information. MS completed data extraction, with 50% also extracted by RM to ensure reliability. Disagreements were discussed and resolved between authors.

### Quality assessment

The Cochrane Risk of Bias 2 (RoB-2) tool [99], using the specific guidance for cluster-randomized trials was used to critically evaluate the methodological quality of the included studies. The measure assesses five areas of potential bias including: (1) randomization (including timing of identification and recruitment of participants); (2) deviations from the intended interventions; (3) missing outcome data; (4) measurement of the outcome; and (5) selection of the reported result. Studies are assessed and shown to have either a low-risk of bias, some concerns relating to bias, or a high-risk of bias, as determined by a validated a priori algorithm. Papers were rated by MS, with 50% of randomly selected papers also evaluated by RM. Disagreements were discussed between authors.

### Effect measures

Where possible, the results section was presented with effect sizes. Given the heterogeneity of papers, a variety of effect size measures were reported. This included Cohen’s *d* (small = 0.2, medium = 0.5, large = 0.8), Hedge’s *g* (small = 0.2, medium = 0.5, large = 0.8), and partial eta squared (small = 0.01, medium = 0.06, large = 0.14).

### Synthesis methods

A meta-analysis was not performed due to substantial heterogeneity across studies in terms of universal population, intervention format, control conditions, and outcome measures. Thus, a narrative synthesis was deemed the most appropriate approach. This review follows the Synthesis Without Meta-Analysis (SWiM) guidelines [14] following the shortcomings of previous narrative syntheses. See Supplementary Materials 3 for SWiM checklist. Findings were grouped according to aims of the review: (1) ED outcomes; (2) physical health outcomes; and (3) broader psychological well-being outcomes. Within this, authors synthesized according to use of individual outcome measure and where possible gender.

**Table 1:** Inclusion and exclusion criteria

	Inclusion	Exclusion
Participants	• Boys or girls aged 11–18 or presented data stratified by age enabling extraction of data from of children 11–18 years. Studies were included if the age range included a small proportion of boys or girls aged 10 or 19 years, but the sample mean, and majority of participants were within 11–18 years. • Boys or girls not identified as having an eating disorder, or at high-risk of developing one.	• Boys and girls under 11 years old, adults, and older adults. • Have an eating disorder or at high-risk of developing one.
Intervention	• Universal (programs target a whole population group that have not been identified as being at risk) secondary school-based interventions. • Interventions must address transdiagnostic core eating disorder psychopathology of weight, shape, or eating concern. • Interventions must include at least one other identified, modifiable psychological risk factor *(e.g.*,* comorbid conditions*,* social media*,* exercise*,* nutrition*,* body image*,* perfectionism*,* self-esteem*,* or mood/well-being)*	• Interventions not based in a secondary school (e.g., primary school or university settings). • Selective or indicated interventions. • Interventions not delivered solely in school lessons (e.g., after school interventions, web-based interventions but recruited from schools). • Specialist institutions (e.g., ballet or sport schools). • Integrated parent and/or teacher interventions. • Interventions that do not address transdiagnostic core eating disorder psychopathology of weight, shape, or eating concern (e.g., obesity interventions). • Interventions that do not address at least one other identified, modifiable psychological risk factor.
Outcomes	• Post-intervention changes measuring transdiagnostic core eating disorder psychopathology outcomes (e.g., EDE-Q) and at least one psychological risk factor (e.g., mood/well-being, self-esteem, social media, exercise, nutrition, perfectionism, comorbid conditions)	• Report solely acceptability or feasibility outcomes.
Study design	• Randomised control trials	• Studies employing other designs, such as observational studies, quasi-experimental studies, or case reports • Qualitative studies • Non-English studies • Book chapter or review articles • Gray literature and unpublished
Comparator	• Any comparison group	

## Results

### Study characteristics

Seventeen RCTs were included in the final analysis (see Table 2). Most studies were cluster RCTs, whereas three out of the 17 were not classified as such [1, 20, 87]. Studies were conducted across eight countries including Spain (*n* = 4), Germany (*n* = 3), Australia (*n* = 3), Italy (*n* = 2), USA (*n* = 1), Argentina (*n* = 1), UK (*n* = 1), and Norway (*n* = 1). One study was conducted in Italy and United States of America (USA). Studies were published between 2001 and 2023.

A total of 11,587 participants were included across studies, ranging from 78 to 2,442 per study. Most studies recruited from state schools, with three from both state and private, and two from private schools only. Two studies did not report school type. Age of individuals ranged from 11 to 18 years old, with the majority being in middle adolescence (*M* = 14.18, *SD* = 0.58). Nine studies were comprised exclusively of girls, eight were comprised of boys and girls. No papers exclusively studied boys. Only half of the studies reported on race and ethnicity, with most participants coming from a Caucasian background (47-55%). Of these studies, some reported birthplace, with a mixture of European, American, and Australian participants. Only one study reported socioeconomic status (SES), with five studies reporting parental income or education. Ten studies reported mean BMI of participants (range 18.3–22.70 kg/m^2^). Outcomes adopted across studies were variable, self-report measures. Duration of follow-up ranged from immediately post-intervention to 2 ½ years post-intervention with the most common at six and twelve-months. Despite universal interventions, three studies [1, 114, 116] utilized ED measures to create low and high-risk groups for facilitation and analysis of data. Additionally, Wilksch et al. [116, 117] used the same dataset from a single RCT.

### Intervention characteristics

All interventions aimed at reducing weight, shape, or eating concerns (see Table 3). Interventions covered a range of risk factors including body image (*n* = 12), social media (*n* = 12), dieting (*n* = 9), exercise (*n* = 8), nutrition (*n* = 8), self-esteem (*n* = 6), emotions (*n* = 5), and perfectionism (*n* = 1). Strategies commonly incorporated in interventions included group discussions (*n* = 12), practical in-class activities (*n* = 7), critical analysis (e.g., actively questioning or critiquing media content) (*n* = 5), videos (*n* = 4), letter/journal writing (*n* = 3), roleplays (*n* = 2), and homework (*n* = 2).

Interventions were commonly delivered in face-to-face group format (*n* = 16), with one study of individual and online format. Psychologists (*n* = 6) and teachers (*n* = 4) facilitated most interventions, with other professionals including research authors and assistants (*n* = 4), postgraduate students (*n* = 2), and coaches (*n* = 1). Intervention length ranged between 2 and 9 sessions (*M* = 5.24, *SD* = 2.14). Most studies offered weekly sessions ranging from 45 to 120 min (*M* = 82.33, *SD* = 30.35). Two studies did not report session lengths. Comparators included active controls, defined as alternative structured activities (including class as usual [CAU]), waitlist control (WLC) groups who received the intervention after the study period, and non-treatment conditions. However, the comparators were not well described across studies.

### Methodological quality assessment

**Fig. 2:**
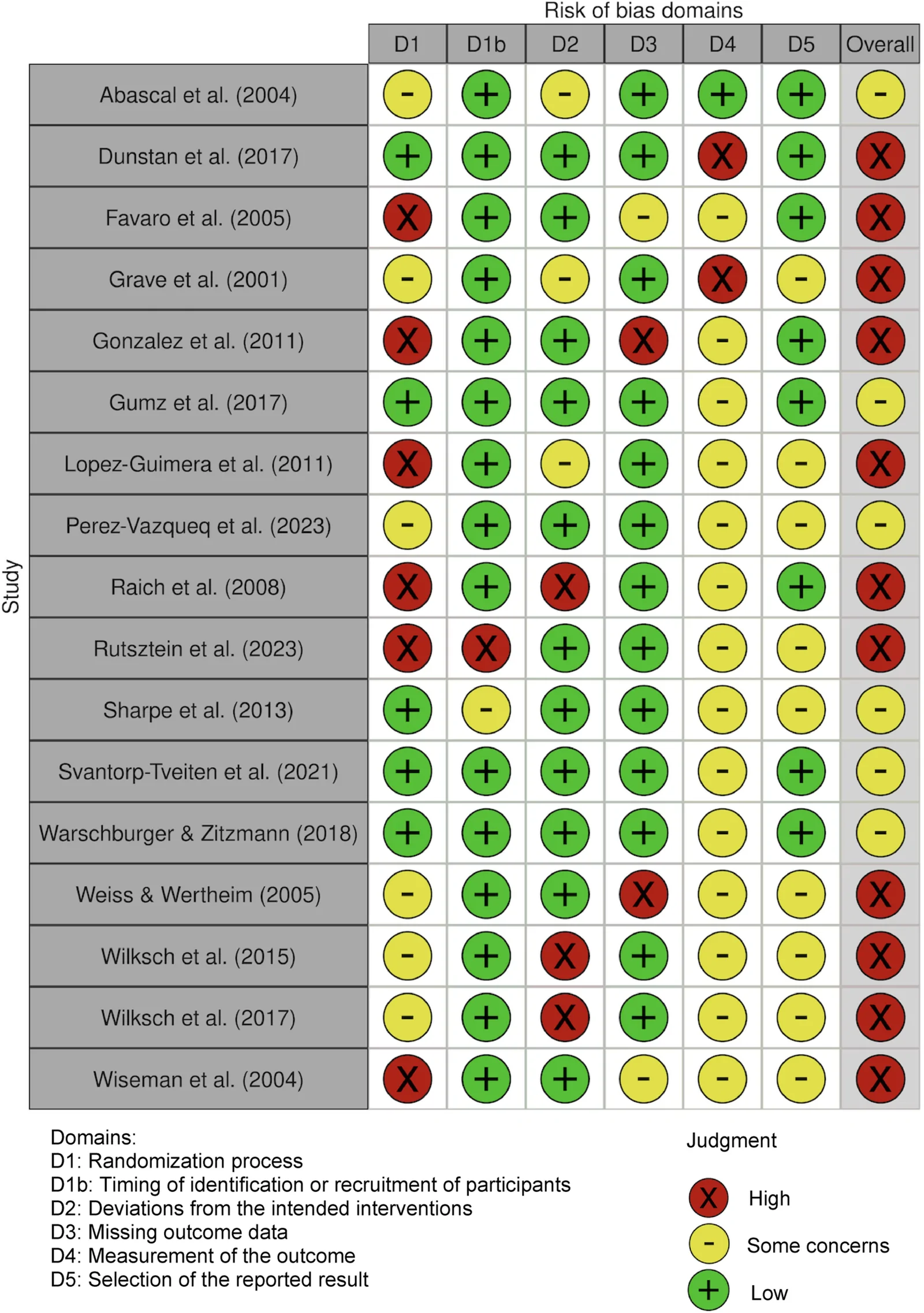
Risk of Bias Domains created using Robvis [69]

Domain scores for RoB-2 in each study are summarised in Fig. 2. Inter-rater reliability was almost perfect κ = 0.824 [70]. Significantly, no included study scored low on RoB-2 overall. Most studies (64.71%) were rated as high-risk overall, with the remaining studies (35.29%) scoring with some concerns. Of the domains, the randomization process was the most problematic, with 6 studies scoring as high-risk, primarily because allocation to the intervention was not random. The timing of recruitment domain, however, received the highest proportion of low-risk ratings (*n* = 15), suggesting recruitment occurred before randomization in most studies.

**Table 2:** Description of randomised controlled trials included in review

Study & country	School setting	*N*	Gender & age	Ethnicity/SES	Physical health (at baseline)	Interventions	Outcomes	Analysis	Time of data collection
Abascal et al. [1] USA	Private high schools for girls	78	Gender: 100% girls Age: *M* ± *SD* = 15.3 ± 0.60, range 13–16 years	Ethnicity: 55% Caucasian, 14% Asian, 14% Multiethnic or other, 12% Hispanic/Latina, 1% African American Family income/education/SES NR	BMI NR	Student Bodies: HRHM group *(8 sessions)* Student Bodies: lower-risk or lower-motivated students *(8 sessions)* Student Bodies: HRHM & lower-risk or lower-motivated students combined *(8 sessions)*	EDE-Q EDI (drive for thinness and bulimic symptoms)	Completer (*N* = 75)	Baseline EOT
Dunstan et al. [22] Australia	State secondary schools	200	Gender: 100% girls Age range: 11–14 years **Co-ed group** Age: *M* ± *SD* = 12.67 ± 0.44 **Single-sex group** Age: *M* ± *SD* = 12.66 ± 0.38 **Control group** Age: *M* ± *SD* = 12.77 ± 0.41	Ethnicity: 90% Australian Family income/education, SES NR	BMI NR	Happy Being Me (co-educational) *6 sessions* Happy Being Me (single sex) *6 sessions* Non treatment condition	EDI-BD SATAQ-I (Internalization-General Media Subscale) PACS RSES MRFS-III (weight teasing & peers subscale) ACS DEBQ-Restraint	ITT	Baseline EOT FU6M
Dalle Grave et al. [20] Italy	State middle school	106	Gender: 57.55% girls, 61, 42.45% boys Age: *M* = 11.60, range 11–12 years	Ethnicity NR SES: 54.7% lower class, 45.3% middle class Family income/education NR	BMI: *M* = 18.3	6 weekly group sessions Control condition^a^	EDE-Q EAT-26 RSES	Completer (*N* = 104)	Baseline EOT FU6M FU12M
Favaro et al. [28] Italy	Vocational Training School	141	Gender: 100% girls Age: *M* ± *SD* = 17.0 ± 1.1, range 16–18 years	Ethnicity, Family income/education, SES NR	BMI NR	6 weekly group sessions Non treatment condition	BMI EAT-26	Completer (*N* = 129)	Baseline EOT
González et al. [37] Spain	State and state-subsidised schools	443	Gender: 57.34% girls, 42.67% boys Age: *M* ± *SD* = 13.50 ± 0.4	Ethnicity: 92.8% European, 4.7% South American, 1.9% Moroccan, 1.2% missing data Family income/education, SES NR	BMI: girls, *M* = 20.9 (*SD* = 3.5); boys, *M* = 20.7 (*SD* = 3.4)	Media Literacy *(4 sessions)* Media Literacy + Nutrition Awareness *(5 sessions)* Non treatment condition	EAT-26 CIMEC-26	Completer (*N* = 276)	Baseline EOT FU7M FU30M
Gumz et al. [42]^b^ Germany	State secondary schools	2342	Gender: 55.60% girls, 44.40% boys Age: *M* ± *SD* = 14.60 ± 0.22, 14 and 17 years old	Family education (SES proxy): 17% low (both parents), 21.8% high (one parent), 61.2% high (both parents) Ethnicity, Family income, SES NR	BMI: *M* = 19.9 (*SD* = 0.17)	3 sessions delivered over 2 weeks Non treatment condition	Ch-EDE-Q SATAQ-G MSCS PHQ-9 GAD-7	Completer (*N* = 1452)	Baseline EOT FU6M
López-Guimerà et al. [62]^c^ Spain	State and public schools	263	Gender: 100% girls Age: *M* ± *SD* = 13.41 ± 0.38	Ethnicity: 89.73% Spanish, 5.32% Latin-American, 2.28% North-African, 1.90% Mixed origins, 0.76% Unspecified Family income/education, SES NR	BMI: *M* = 21.19 (*SD* = 3.8)	Media Literacy *(4 sessions)* Media Literacy + Nutrition *(5 sessions)* Class as usual *(1 session)*	BMI EAT-26 CIMEC-26	Completer (*N* = 252)	6 Baseline EOT FU6M
Pérez-Vázquez et al. [87] Spain	State secondary schools	161	Gender: 45.96% girls, 54.04% boys **Intervention Group** Age: *M* ± *SD* = 12.46 ± 0.48 **WLC Group** Age: *M* ± *SD* = 12.51 ± 0.48	Ethnicity, Family income/education, SES NR	**Intervention Group** BMI: *M* = 22.40 (*SD* = 4.40) **Control Group** BMI: *M* = 20.60 (*SD* = 3.86)	e-Health psychoeducational intervention *(3 sessions)* WLC	EAT-26 BMI RSES	ITT	Baseline EOT
Raich et al. [89]^c^ Spain	Public and public subsidized private secondary schools	349	Gender: 100% girls Age: *M* ± *SD* = 13.11 ± 0.48	Ethnicity, Family income/education, SES NR	BMI: *M* = 21.57 (*SD* = 4.2)	AMC + ML *(2 sessions)* AMC + ML + NUT *(2 sessions)* Class as usual *(2 sessions)*	EAT-26 CIMEC-26 NUT-Q	Completer (*N* = 288)	Baseline EOT FU6M
Rutsztein et al. [93] Argentina	Secondary schools	509	Gender: 100% girls Age: *M* ± *SD* = 15.39 ± 1.30, range 13–18 years	Ethnicity, Family income/education, SES NR	BMI: *M* = 22.70 (*SD* = 4.37)	PIA-2 *(3 sessions)* Class as usual *(3 sessions)*	EDI-3-RF SATAQ-4 (thin-ideal internalization subscale)	ITT	Baseline EOT FU3M
Sharpe et al. [96] UK	State secondary girl schools	448	Gender: 100% girls Age range: 12–14 years **Intervention Group** Age: *M* ± *SD* = 13.06 ± 0.59 **Control Group** Age: *M* ± *SD* = 12.99 ± 0.54	**Intervention group** Ethnicity: 53% White, 17% Asian, 15% Black, 14% Other Family education (SES proxy): 1% primary education, 24% secondary education, 76% university education **Control group** Ethnicity: 47% White, 21% Black, 19% Asian, 12% Other Family education (SES proxy): 23% secondary education, 78% university education Family income & SES NR	BMI NR	Me, You, & Us *(6 sessions)* Class as usual *(6 sessions)*	BES EDDS (frequencies of binge eating and compensatory behaviors) SATAQ-3 (thin-ideal internalization subscale) ACFS MSPSS (Friends subscale) DASS (Depression subscale)	ITT	Baseline EOT FU3M
Svantorp-Tveiten et al. [104]^d^ Norway	State and private schools	2442	Gender: 33.91% boys, 66.09% girls Age range: 16–18 years **Intervention Group** Age boys: *M* ± *SD* = 16.77 ± 0.46 Age girls: *M* ± *SD* = 16.76 ± 0.49 **Control Group** Age boys: *M* ± *SD* = 16.73 ± 0.49 Age girls: *M* ± *SD* = 16.78 ± 0.48	**Intervention Group** Parental income (boys): >$104,000 48.80% Parental income (girls): >$104,000 34.90% Family education (SES proxy): boys: 76.80% paternal university education, 79.80% maternal university education; girls: 72.90% paternal university education, 74.80% maternal university education **Control Group** Parental income (boys): >$104,000 44% Parental income (girls): >$104,000 25.50% Family education (SES proxy): boys: 64% paternal university education, 64.60% maternal university education; girls: 63.40% paternal university education, 70.80% maternal university education Ethnicity & SES NR	**Intervention Group** BMI: girls, *M* = 21.41 (*SD* = 2.79); boys, *M* = 21.58 (*SD* = 2.64) **Control Group** BMI: girls, *M* = 21.48 (*SD* = 3.11); boys, *M* = 21.84 (*SD* = 3.16)	Healthy Body Image *(3 sessions)* Class as usual *(3 sessions)*	EDE-Q RSES SCL-10 BIAAQ SATAQ-4 (media pressure, thin & muscular internalization subscales) DLS Protein, creatine and diet aids use (weekly frequency)	ITT (*N* = 1228)	Baseline EOT FU3M FU12M
Warschburger and Zitzmann [112] Germany	Grammar and comprehensive schools	1112	**Intervention Group** Gender: 52% girls, 48% boys Age: *M* ± *SD* = 13.0 ± 0.80, range 11–16 years **Control Group** Gender: 56% girls, 44% boys Age: *M* ± *SD* = 12.90 ± 0.80, range 10–16 years	Ethnicity, Family income/education, SES NR	**Intervention** BMI: *M* = 18.9 (*SD* = 2.8) **Control** BMI: *M* = 18.4 (*SD* = 2.4)	Potsdam Prevention at Schools *(9 sessions)* WLC	EAT-26 EDI-2 (drive for thinness and bulimic symptoms) SATAQ-3 (internalization and media pressure subscales) OEQ PACS POTS	ITT	Baseline EOT FU12M
Weiss and Wertheim [114] Australia	Catholic private girl schools	173	Gender: 100% girls Age: 47% 14 -14.9 years, 50% 15 -15.9 years, 3% 16-16.9 years	Ethnicity: 83% Australian, 9% Asian, 9% European Family income /education, SES NR	BMI NR	*4 weekly group sessions* Non treatment condition	RSES EDI (drive for thinness, bulimic symptoms, body dissatisfaction and interoceptive awareness) WLBS (Food abstinence & Purging)	NR	Baseline EOT FU3M
Wilksch et al. [116]^e^ Australia	Public, private, and Catholic schools	1316	Gender: 64% girls, 36% boys Age: *M* ± *SD* = 13.21 ± 0.68	Ethnicity: 26.97% South Australian, 35.49% Victoria, 37.54% Western Australia Family income/education, SES NR	BMI NR	Media Smart *(8 sessions)* Life Smart *(8 sessions)* HELPP *(8 sessions)* Class as usual	EDE-Q (weight, shape, & eating concerns subscales) DEBQ- Restraint EDI-BD SATAQ-3 (media internalization subscale) PSPS CDI MRFS-III MPS Project EAT Physical activity (mean hours per day)	Completer (*N* = 941)	Baseline EOT FU6M FU12M
Wilksch et al. [117]^e^ Australia	See Wilksch et al. [116]								
Wiseman et al. [118] Italy and USA	High schools	188	Gender: 100% girls Age: range 15–16 years	Ethnicity, Family income/education, SES NR	BMI: USA only, *M* = 20.85 (*SD* = 2.77)	*5 group sessions* Health lectures *(5 sessions)*	RSES EDI TFEQ	NR	Baseline EOT

ACFS: Appearance Conversations with Friends Scale; ACS: Appearance Conversations Scale; AMC: Aesthetic Model Criticism; BES: Body Esteem Scale for Adults and Adolescents; BIAQ: Body Image Avoidance Questionnaire; BIAAQ: Body Image–Acceptance and Action Questionnaire; BMI: Body Mass Index; BSQ-8c: Body Shape Questionnaire–Short Version; CDI: Child Depression Inventory; Ch-EDE-Q: Children’s version of the Eating Disorder Examination Questionnaire; CIMEC-26: Questionnaire on Influences of Aesthetic Body Ideal-26; DASS: Depression, Anxiety, and Stress Scale; DEBQ: Dutch Eating Behavior Questionnaire; DLS: Drive for Leanness Scale; EAT-26: Eating Attitude Test; EDDS: Eating Disorder Diagnostic Scale; EDE-Q: Eating Disorders Examination Questionnaire; EDI: Eating Disorder Inventory; EDI-2: Eating Disorder Inventory-2; EDI-3-RF: Eating Disorder Inventory-3–Eating Disorder Risk Index; EDI-BD: Eating Disorder Inventory–Body Dissatisfaction Scale; EOT: end of treatment; FU: follow-up; GAD-7: Generalized Anxiety Disorder Scale; HELPP: Helping, Encouraging, Listening and Protecting Peers Initiative; HRHM: higher-risk & higher-motivated; ITT: Intention to treat; M: Mean; ML: Media Literacy; MPS: Multidimensional Perfectionism Scale; MRFS-III: McKnight Risk Factor Survey; MSCS: Multidimensional Self-Concept Scale; MSPSS: Multidimensional Scale of Perceived Social Support; NR: not reported; NUT: Nutrition; NUT-Q: Nutrition Questionnaire; OEQ: Obligatory Exercise Questionnaire; PACS: Physical Appearance Comparison Scale; PHQ-9: Patient Health Questionnaire-9; PIA-2: prevenci onintegrada de trastornos alimentario y obesidad [Integrated prevention of eating disorders and obesity]; POTS: Perception of Teasing Scale; Project EAT: Project Eating Among Teens; PSPS: Perceived Sociocultural Pressure Scale; RSES: Rosenberg Self-Esteem Scale; SATAQ-G: Sociocultural Attitudes Towards Appearance Questionnaire; SATAQ-I: Sociocultural Attitudes Toward Appearance Scale-3; SATAQ-4: Sociocultural Attitudes Towards Appearance Questionnaire-4; SCL-10: Hopkins Symptom Checklist-10; SD: Standard Deviation; TFEQ: Three Factor Eating Questionnaire; WLBS: Weight Loss Behaviors Scale; WLC: waitlist control

^a^Comparator condition is unclear. Authors were emailed but reviewers did not receive a reply

^b^Demographics are recorded from completers

^c^Only female results have been analyzed

^d^Demographics for participants included in the effect analyzes

^e^Demographic information and study description compiled for all three studies as they report findings from the same randomised control trial

**Table 3:** Details of interventions and outcomes of Randomised controlled trials

Study	Descriptions of interventions core psychopathology/risk factors addressed	End-of-treatment	Follow-up
Abascal et al. [1]^a^	**Name of intervention**: Student Bodies **Duration**: 8 × 45 min individual sessions over 6 weeks **Content**: Readings addressing issues such as ***body image dissatisfaction***, ***excessive weight concerns***, ***dieting*** or restrained eating patterns, ***exercise*** and ***nutrition***. This was followed by a cognitive task (e.g., challenging negative thoughts and observing ‘real’ women in public, and comparing them to women seen in fashion magazines). Students encouraged to post weekly entries in the confidential body image journal, with the goal for students to identify the situations that trigger their negative body image and to eventually challenge their dysfunctional beliefs. **Delivery**: Completed online in computer labs. Students logged onto the program during class. **Control**: Student Bodies (HRHM & lower-risk or lower-motivated students combined)	*Primary outcomes* HRHM-C participants saw a significant improvement in ED psychopathology of restraint (*p* < .050, Effect Size^b^ = 0.49), shape concern (*p* < .010, Effect Size = 0.75), drive for thinness (*p* < .050, Effect Size = 0.75), but not in bulimic symptoms. Other-C participants saw no significant improvements in drive for thinness or bulimic symptoms.	No follow-up.
Dunstan et al. [22]^c^	**Name of intervention**: Happy Being Me **Duration**: 6 x group sessions over 6 weeks. Length of sessions unknown. **Content**: *Session 1*: introduction to intervention and identify and challenge sociocultural appearance ideals. *Session 2*: Identify the occurrence and consequences of appearance teasing and focus on qualities other than appearance. *Session 3*: Impact of appearance conversations (emphasis on ***weight and shape***) and develop alternatives to engaging in appearance conversations. *Session 4*: understand the concept of appearance comparisons and the negative influence this can have on ***body image***. *Session 5*: explore and challenge the relationship between the ***media*** and beliefs around appearance. *Session 6*: review and consolidate important messages of each session. **Delivery**: Facilitated by two psychology research assistants. Overview of the topic and group discussion **Control**: Non-treatment condition	*Primary outcomes* Intervention group saw a significant improvement in body dissatisfaction at post-treatment (*p* < .010, *η*^*2*^ = 0.030) compared to the control group. *Secondary outcomes* Dietary restraint improved over time regardless of having the intervention or not (*p* = .010, *η*^*2*^ = 0.06). Dietary restraint did not change over time between the intervention or control group (*p* = .310). Significant interaction effect for self-esteem (*p* = .020, *η*^*2*^ = 0.04), with compared to the control group, self-esteem was significantly higher for participants in the intervention group post-intervention (*p* < .010). Significant interaction effect for internalization of the thin ideal (*p* = .010, *η*^*2*^ = 0.06) with scores significantly lower post-intervention in the intervention group compared to the control group (*p* < .010, *d* = 0.26). Significant interaction effect for appearance comparisons (*p* = .010, *η*^*2*^ = 0.04) with scores significantly lower post-intervention in the intervention group compared to the control group (*p* < .010). Appearance conversation scores improved over time regardless of having the intervention or not (*p* = .040, *η*^*2*^ = 0.03), but not between the intervention or control group (*p* = .140). Weight-related teasing scores did not change over time between the intervention or control group (*p* = .390).	*Primary outcomes* Body dissatisfaction was not significantly different at follow-up in the intervention group. *Secondary outcomes* Compared to the control group, self-esteem (*p* < .050) and appearance comparisons (*p* < .010) were significantly higher for participants in the intervention group at follow-up, with internalization of the thin ideal significantly lower for participants in the intervention group at follow-up (*p* < .010) compared to the control group.
Dalle Grave et al. [20]	**Duration**: 6 × 2 h group sessions over 6 weeks **Content**: *Aim 1*: increase knowledge on sociocultural ***pressure to be thin***, cognitive distortions (e.g., extreme concern for ***shape and body weight***, ***low self-concept***, ***perfectionism***, and dichotomous thinking), effects of ***dieting*** and EDs. *Aim 2*: help avoid developing an ED by reducing the importance of shape and body weight in self-evaluation. **Delivery**: Facilitated by two psychologists. First 30 min were dedicated to the educational materials, the remaining time was devoted to practical activities, group discussions, and revision of homework. **Control**: Comparator condition is unclear	*Primary outcomes* Global EDE-Q scores significantly decreased over time in both groups (*p* < .050). Eating concerns in the intervention group significant decreased post-intervention compared to the control group (*p* < .010). EAT scores significantly decreased over time in both conditions (*p* < .010). Scores decreased significantly post-intervention in the intervention group compared to the control group (*p* < .010). *Secondary outcomes* Self-esteem scores changed significantly over time (*p* < .010) without significant differences between groups.	*Primary outcomes* No significant differences in EAT scores, global or subscale EDE-Q between groups at either 6-month or 12-month follow-up.
Favaro et al. [28]	**Duration**: 6 × 2 h group sessions over 6 weeks **Content**: *Session 1*: relation between adolescents and their family, adolescents, their peers, with themselves, and their own body. *Session 2*: the body during adolescence, with descriptions of normal growth and development processes. Concepts like ideal body and body ideal, body acceptance, ***body dissatisfaction***, and body denigration were also discussed. *Session 3*: dealing with the media’s overemphasis on thinness and to discourage the use of excessive caloric restriction. ***Pressure to be thin***, different methods to lose weight, advantages and disadvantages of ***dieting***, effects on normal development, reasons for the failure of excessive dieting, and the potential link with the development of EDs. *Session 4*: about eating disorders, their diagnostic criteria, clinical features, and treatments. Session 5: prevention of EDs. *Session 6*: ***media*** and its impact on their own beliefs about beauty, thinness, fitness, health, and well-being. **Delivery**: Facilitated by teachers. Overview of the topic, group discussions, written summary post session **Control**: Non-treatment condition	*Primary outcomes* Intervention group showed a significant improvement in bulimia symptoms (*p* < .005) compared to no change observed in control group. No significant differences were observed between groups for total EAT, or the subscales of dieting and oral control. No significant differences were observed in either intervention or control group for BMI (*p* > .050).	No follow-up.
González et al. [37]	**Name of intervention**: ML and ML + NUT **Duration**: 2 h weekly group sessions *(ML = 4 sessions; NUT + ML = 5 sessions).* Length of sessions unknown. **Content**: *ML components*: beauty throughout history, recent changes in the criteria for beauty, sizes of models, ***thinness*** of the West, ***advertising messages***, real effectiveness of ***diets*** and their effects, manipulation of advertisements, association between beauty and happiness, and ***real women: a variety of sizes***. *NUT components*: definition of balanced eating, ***nutrition*** and eating, nutrients, Food Pyramid and foods, water, analysis of menus, and final recommendations. **Delivery**: Facilitated by authors of program. Interactive format with a PowerPoint multimedia display, group discussions, and letter writing. Two activities and tasks between sessions. **Control**: Non-treatment condition	*Primary outcomes* Participants in ML group (*p* = .009) and ML + NUT (*p* = .002) group showed significantly lower ED pathology scores than the control group. No differences between ML and ML + NUT groups on ED pathology. *Secondary outcomes* Participants in the ML group (*p* = .001) and ML + NUT (*p* = .004) showed significantly lower CIMEC scores than the control group. No differences between ML and ML + NUT groups on CIMEC. Post-treatment ML participants displayed lower scores than the control group for CIMEC, whereas ML + NUT participants did not differ from the control group.	*Primary outcomes* ML group scored lower than the control group for ED pathology and CIMEC at both 7-month (ED; *p* = .025, CIMEC; *p* < .001) and 30‐month (ED; *p* = .002, CIMEC; *p* = .016) follow‐up assessments. ML + NUT group scored lower than the control group for ED pathology and CIMEC at both 7-month (ED; *p* = .022, CIMEC; *p* = .001) and 30‐month (ED; *p* < .001, CIMEC; *p* = .037) follow‐up assessments.
Gumz et al. [42]^d^	**Duration**: 3 × 2 h group sessions over 2 weeks **Content**: *Session 1*: origin and maintenance of western beauty ideals, costs and benefits of these ideals, and strategies for coping with pressure from family, peers, and media. *Session 2*: ***body perception***, sources of ***self-esteem***, consequences of being ***under/overweight***, healthy ***nutrition***, coping with *peer pressure*, ***healthy physical exercise***, self-expression in ***social media***, coping with ***negative emotions***,*** and stress***. *Session 3*: information on EDs including risk factors and symptoms, treatment options, coping with ED peers, and competence for help-seeking. **Delivery**: Facilitated by 1 member of the Center of the Prevention of Addiction, and 1 member of the research team. Overview of the topic, group discussions, individual reflections, interactive interventions, nine station parcour (session 2), and videos. **Control**: Non-treatment condition	*Primary outcomes* No significant differences were observed in intervention or control group in ED pathology from baseline to post-treatment (*p* = .410). Significant interaction (gender x time x grade) indicated that in both groups, 8th grade females exhibited increased ED pathology post-intervention, whereas in 8th grade males, the risk decreased post-intervention (interaction *p* < .001). *Secondary outcomes* Intervention group showed significantly greater improvements in anxiety symptoms over time (*p* < .010). Post-treatment participants showed a greater reduction in anxiety (*p* = .020, *d* = 0.11) compared to the control group. The intervention group showed significantly greater increase in experienced pressure due to internalization of Western beauty ideals post-intervention (*p* <. 010, *d* = 0.04) compared to the control group. Depression (*p* = .640), self-concept (*p* = .370), internalization of body ideals (*p* = .100), and awareness of body image (*p* = .118) did not significantly change over time between the intervention or control group.	*Primary outcomes* No significant differences were observed in intervention or control group in ED pathology from baseline to follow-up. Significant interaction (group x time x grade) (*p* = .049) with both 11th grade boys and girls in the intervention group exhibiting lower ED pathology than those in the control group at 6-month follow-up. While 8th grade boys and girls showed the opposite trends (interaction *p* = .012). Significant interaction (gender x time x grade) indicated that in both groups, 8th grade females exhibited increased ED pathology at follow-up, whereas in 8th grade males, the risk remained stable (interaction *p* < .001). At 6-months follow-up, there was no significant difference in anxiety (*p* = .510) or experienced pressure due to internalization of Western beauty ideals (*p* = .300) between conditions.
López-Guimerà et al. [62]	**Name of intervention**: ML and ML + NUT **Duration**: *ML*: 2 × 90-min group sessions + 2 × 60-min activity group sessions. *NUT + ML*: 1 × 90-min group session) **Content**: *ML Session 1*: feminine beauty ideal, ***thinness*** in Western culture, and the beauty ideal in the media. *ML Session 2*: advertising analysis. *ML Session 3*: how to deal with ***media*** messages. *ML Session 4*: complaint letters to the media. *NUT Session*: balanced eating concept, the concepts of eating and ***nutrition***, nutrients, the Food Pyramid and foods, the importance of water, and analysis of diet **Delivery**: Facilitated by two authors of the intervention. The two activities were facilitated by teachers. Overview of the topic, critical analysis, group discussion, and letter writing. **Control**: Non-treatment condition	*Primary outcomes* Only EAT diet subscale showed effects between conditions. Median of change for the diet subscale in the NUT + ML group is significantly higher post-treatment than the median of change for the non-treatment group (*p* < .050, *d* = 0.37). No significant differences were observed in interventions or control group for BMI post-intervention.	*Primary outcomes* Median of change for the diet subscale in the ML group is significantly higher at follow-up than the median of change for the non-treatment group (*p* < .050, *d* = 0.37). No significant differences were observed in interventions or control group for BMI at 6-month follow-up.
Pérez-Vázquez et al. [87]	**Name of intervention**: e-Health psychoeducation **Duration**: 3 × 55 min group sessions over 2 weeks **Content**: *Session 1*: promoting healthy eating attitudes and behaviors. ***Dieting***,*** fasting***, and the risks involved. *Session 2*: benefits of regular activity for better health, and how activity improves our body satisfaction, and how it reflects on ***self-esteem***. Sleep hygiene. *Session 3*: discussing body ideals, the wide range of different body shapes during adolescence, the development of coping skills to address sociocultural ***pressures to be thin***, and to critically assess ***media*** and ***societal attitudes about body shape and weight*** **Delivery**: Facilitated by teachers but intervention was entirely online. Modules accessible 24/7 via Moodle platform (for 3 months). Each month a module was unlocked, and students had set class period to engage with module activities including videos, and knowledge questionnaires. **Control**: WLC	*Primary outcomes* Intervention group saw significant reductions in total EAT (*p* < .050, *d* = 0.32) and oral control subscale (*p* < .050, *d* = 0.33) compared to WLC. No significant differences by sex between groups on ED symptomatology. No significant differences were observed in the intervention or WLC group for BMI. *Secondary outcomes* No significant differences were observed in the intervention or WLC group for self-esteem.	No follow-up.
Raich et al. [89]^e^	**Name of intervention**: AMC + ML and AMC + ML + NUT **Duration**: 2 × 90 min group sessions over 2 weeks **Content**: *NUT Session*: definition of balanced eating, definition and differentiation between ***nutrition*** and eating, nutrients, Food Pyramid and foods, water, and analysis of menus. *AMC + ML Session*: beauty throughout history, recent changes in the criteria for beauty, sizes of models, ***thinness*** of the West, ***advertising messages***, real effectiveness of ***diets*** and their effects, manipulation of advertisements, association between beauty and happiness, and ***real women: a variety of sizes***. **Delivery**: Facilitated by 2 coaching members of the team. Psychoeducational program including group debate, and visual presentations. **Control**: Class as usual	*Primary outcomes* No significant differences between groups at post-intervention on total EAT (*p* = .257), or subscales of diet (*p* = .053), bulimia (*p* = .838), or oral control (*p* = .973). *Secondary outcomes* Significant group x time effect for nutritional knowledge (*p* < .001), with knowledge significantly increasing for participants in the AMC + ML + NUT condition compared to the control group (*g* = 0.60).	*Primary outcomes* No significant differences between groups at follow-up on total EAT (*p* = .368), or subscales of diet (*p* = .839), bulimia (*p* = .373), or oral control (*p* = .296). *Secondary outcomes* No statistically significant differences in knowledge of nutrition between groups (*p* = .322).
Rutsztein et al. [93]^f^	**Name of intervention**: PIA-2 **Duration**: 3 × 90 min group sessions over 3 weeks **Content**: *Session 1*: critical analysis of magazine ads and Instagram images, the physical consequences of trying to achieve a ***thin ideal***, and coping strategies against social pressure towards thinness. *Session 2*: food groups and the importance of including them in meals, and the advantages of avoiding ***restrictive diets*** and regular meal skipping. *Session 3*: discussions around ***physical activity***. **Delivery**: Facilitated by 2 authors of the paper. Overview of the topic, critical analysis, videos, group discussions, role-play activities, and games. **Control**: Class as usual	*Primary outcomes* A significant effect of time on ED risk (*p* < .001; ICC = 0.04). On average, participants decreased 0.42 units of ED risk month by month. No significant differences were observed in intervention or control group for ED risk over time, when controlling for age (*p* = .980). *Secondary outcomes* Thin-ideal internalization changed significantly over time (*p* <. 001; ICC = 0.03). Thin-ideal internalization did not significantly change month by month between the intervention or control group (*p* = .520).	
Sharpe et al. [96]	**Name of intervention**: Me, You, & Us **Duration**: 6 × 50 min group sessions over 6 weeks **Content**: *Session 1*: definition of beauty and how ideas about beauty are shaped by images in the media. *Session 2*: negative effects of ***media*** on body confidence. *Session 3*: why people tend to ***‘fat talk’*** and their effects. *Session 4*: reducing the amount of fat talking adolescents engage in. *Session 5*: personal strengths and how to put personal strengths to better use. *Session 6*: happiness and where it comes from. **Delivery**: Facilitated by teachers. Overview of the topic, group discussions, in-session activities, power point presentations, and videos. **Control**: Class as usual	*Primary outcomes* No significant differences were observed in intervention or control group for binge eating (*p* = .230) or compensatory behaviors (*p* = .220). *Secondary outcomes* No significant differences were observed in intervention or control group for depression (*p* = .500), peer support (*p* = .400), appearance conversations (*p* = .900), or body esteem (*p* = .070). Girls in the clinical range for body esteem showed greater reliable change in the direction of improvement in the intervention group (32%) compared with the control group (7%) (*p* = .020). This was the same for girls in the normal body esteem range (*p* = .010) Thin-ideal internalization scores were significantly lower in the intervention group compared to the control group post-intervention (*p* = .040, *d* = 0.17).	*Secondary outcomes* Thin-ideal internalization scores were significantly lower in the intervention group compared to the control group at follow-up (*p* = .050, *d* = 0.16). Girls in the intervention group had significantly higher body esteem compared to the control group at follow-up (*p* = .006).
Svantorp-Tveiten et al. [104]	**Name of intervention**: Healthy Body Image **Duration**: 3 × 90 min group sessions over 9 weeks **Content**: *Session 1*: body image awareness and ***‘fat talk’***, peer and family influences, appearance and social comparison, ***self-esteem***, and ***social media***. *Session 2*: how to make social media a positive experience and how adolescents influence their peers on social media. *Session 3*: ***exercise***, supplement use, and ***nutrition***. **Delivery**: Facilitated by 2 clinical psychologists. Overview of the topic, presentations, and classroom activities. **Control**: Class as usual	*Primary outcomes* Group x gender interaction on ED symptomatology (*p* = .048). Girls benefitted from the intervention (*p* = < 0.001, *d* = 0.21) whereas boys did not (*p* = .470, *d* = 0.05). No difference in ED symptomatology post-intervention between groups (*p* = .093). *Secondary outcomes* Intervention group anxiety & depression scores maintained over time (*p* < .001, *d* = 0.20) whilst the control group scores increased. There were no significant differences in anxiety & depression scores post-treatment (*p* = .993). Self-esteem significantly differed over time between groups (*p* = .022). Compared to the intervention group the control group saw a significant decrease in self-esteem post-intervention (*p* = .020, *d* = 0.15). The intervention group had significantly lower drive for leanness scores than the control group at all measurement time points (*p* = .003, *d* = 0.12). The intervention group had significantly higher body image flexibility than the control group at all measurement timepoints (*p* < .001). A significant group x time x gender effect for body image flexibility (*p* = .011). Girls from the intervention group significantly increased body image flexibility posttreatment compared to control girls (*p* = .024, *d* = 0.18). The intervention group reported significantly lower media pressure than the control group at all measurement time points (*p* <. 001). Significant group x gender interaction with girls reporting significantly lower media pressure than boys at all measurement time points (*p* = .036). The intervention group reported significantly lower thin internalization than the control group at all measurement time points (*p* <. 001). Significant group x gender interaction (*p* = .040) with girls reporting significantly lower thin internalization than boys at all measurement time points (*p* <. 001, *d* = 0.24). The intervention group reported significantly lower muscular internalization than the control group (*p* = .001, *d* = 0.15). No main or interaction effects were observed for diet aid use.	*Primary outcomes* The intervention group scored significantly lower than the control group at both 3-month (*p* = .022, *d* = 0.17) and 12-month (*p* < .001, *d* = 0.27) follow-ups. *Secondary outcomes* The intervention group showed significantly greater improvements in anxiety and depression symptoms (*p* < .001, *d* = 0.31), and self-esteem (*p* < .001, *d* = 0.33) at 12-month follow-up, but not 3-months compared to the control group. Girls from the intervention group significantly increased body image flexibility at 12-month follow-up compared to control girls (*p* < .001, *d* = 0.47). Control girls’ body image flexibility significantly decreased between 3-month and 12-month follow-up (*p* = .004, *d* = 0.21). Whilst boys in the intervention group significantly increased body image flexibility at 3-month follow-up compared to control boys (*p* = .003, *d* = 0.34). Thin internalization effect among girls sustained across 3-month (*p* <. 001, *d* = 0.40) and 12-month follow-ups (*p* <. 001, *d* = 0.44). Weekly frequency of protein supplement usage in intervention boys remained stable, whilst it increased for control boys at 3-month (*p* < .001, *d* = 0.50) and 12-month (*p* =. 020, *d* = 0.43) follow-ups. Weekly frequency of creatine supplement usage increased for control boys at 3-month (*p* =. 003, *d* = 0.40) and 12-month (*p* =. 045, *d* = 0.32) follow-ups.
Warschburger and Zitzmann [112]	**Name of intervention**: Potsdam Prevention at Schools **Duration**: 9 × 45 min group sessions over 9 weeks **Content**: *Session 1*: healthy ***nutrition***,*** physical exercise***, reflection of own eating habits, and reduction of myths about eating, and ***dieting***. *Session 2*: promotion of a critical distance from beauty ideals in ***media***. *Session 3*: critical reflection of ***slimness ideal***, recognition of the diversity and multidimensionality of the concept of attractivity and self-concept, and discussion of the importance of one’s own appearance for the ***self-esteem***. *Session 4*: acceptance of own strengths and weaknesses reflection of the link between general well-being, body acceptance, and enhancing self-esteem. *Session 5*: deepening of knowledge about ***stress*** and coping, checking the appropriateness of own coping strategies, and discovery of appropriate strategies. *Session 6*: discovery and application of steps to solve problems. *Session 7*: sensitisation for motives and consequences of teasing. *Session 8*: individual empowerment in dealing with teasing and peer pressure. *Session 9*: consolidating knowledge, and self-esteem stabilisation. **Delivery**: Facilitated by teacher. Overview of the topic, playful learning, group discussions, critical reflection, in-session and home-based activities. **Control**: WLC	*Primary outcomes* No significant differences were observed in intervention or control group at post-intervention for ED pathology (*p* = .093, *d* = 0.11), drive for thinness (*p* = .090, *d* = 0.11) or bulimic symptoms (*p* = .478). Over time girls in the intervention group saw a significant reduction in both ED pathology (*p* = .006, *d* = 0.23) and bulimic symptoms (*p* = .017, *d* = 0.17). *Secondary outcomes* No significant differences were observed in intervention or control group for perceived weight-related teasing (*p* = .462), thin-ideal internalization (*p* = .085), or perceived teasing (*p* = .462). No gender differences. Significant interaction observed for the emotional element of exercise (*p* = .005), with values significantly reducing in the intervention group post-intervention (*p* = .005, *d* = 0.17) compared to the control group. Significant interaction observed for perceived media pressure (*p* = .008), with values significantly reducing in the intervention group post-intervention (*p* = .012, *d* = 0.16) compared to the control group where values increased. No gender differences. Significant interaction observed for social comparison (*p* = .005) between conditions and over time.	*Primary outcomes* No significant differences were observed in intervention or control group for ED pathology (*p* = .038, *d* = 0.13), drive for thinness (*p* = .090, *d* = 0.11), or bulimic symptoms (*p* = .090, *d* = 0.11) at 12-months follow-up. *Secondary outcomes* The emotional element of exercise significantly reduced in the intervention group at follow-up (*p* = .007, *d* = 0.16). Perceived media pressure significantly reduced in the intervention group at follow-up (*p* = .009, *d* = 0.16) compared to the control group where values increased. Social comparison remained stable in the intervention group at follow-up, whilst scores continuously increased in the control group (*p* = .024, *d* = 0.14).
Weiss and Wertheim [114]	**Duration**: 4 × 100 min group sessions over 4 weeks **Content**: *Session 1*: **body image** or ***eating problems*** and introduction to ***self-esteem***. *Session 2*: feeling good about your body and develop strategies to manage situations that can have a negative effect on body/self-esteem. *Session 3*: dangers of EDs and how to recognize onset of problems. ***Dieting*** and what a healthy lifestyle and healthy eating. *Session 4*: emotional eating, management of ***emotions*** effectively, impact of peers, particularly boys on body-esteem, and of program celebration with sharing of healthy foods and inspirations. **Delivery**: Facilitated by psychologist (first author). Group rules discussed, overview of the topic, group discussions, and small group activities. **Control**: Non treatment condition	*Primary outcomes* High-risk intervention group saw significant improvements in body dissatisfaction (*p* < .010), drive for thinness (*p* < .001), and interoceptive awareness (*p* < .002) compared to low-risk intervention group. High-risk intervention group saw significant improvements in drive for thinness (*p* < .020) compared to low-risk control group and interoceptive awareness (*p* < .010) compared with high-and-low risk control groups. *Secondary outcomes* No significant main effect of risk status (*p* = .090), group (*p* = .930), or group x risk status interaction (*p* = .080) on food abstinence, purging, pressure to be thin, or self-esteem outcomes post-intervention.	*Primary outcomes* No significant interaction (group x risk) on any EDI outcomes at follow-up. *Secondary outcomes* Significant effect of risk status on food abstinence (*p* = .020) and pressure to be thin (*p* = .030), with high-risk group improving significantly more than the low-risk group in both intervention and control groups at follow-up. No effect on purging between conditions at follow-up (*p* > .150). No significant difference in self-esteem between groups at follow-up.
Wilksch et al. [116]	**Name of intervention**: Media Smart, Life Smart, and HELPP **Duration**: 8 × 50 min group sessions over 4 weeks **Content**: *Media Smart Session 1*: magazines and stereotypes. *Session 2*: ***media*** advertising. *Session 3*: who places pressure on us and what can we do about it. *Session 4*: looking after ourselves and our peers. *Session 5*: putting pressure on the media. *Sessions 6 & 7*: is advertising harmful. *Session 8*: how to deal with media. *Life Smart Session 1*: health. *Session 2*: physical health and ***nutrition***. *Session 3*: sleep and ***exercise***. *Session 4*: identifying unhelpful thinking styles. *Session 5*: ***emotions***. *Session 6*: making connections with safe people. *Session 7: prepare for presentation* ‘If someone is ***overweight***, they are unhealthy–agree or disagree? *Session 8*: bringing it all together and looking to the future. *HELPP Session 1*: appearance ideals. *Session 2*: appearance pressure and teasing. *Sessions 3 & 4*: introduction to ***‘fat-talk’*** (appearance conversations) and their negative consequences. *Session 5*: media literacy and ***media pressure***. *Session 6*: appearance comparisons. *Session 7*: ***eating concerns***. *Session 8*: review and challenging appearance ideals. **Delivery**: Facilitated by postgraduate psychology students. Overview of the topic, videos, group discussions, presentations (by students), and role plays. **Control**: Class as usual	*Primary outcomes* Significant group × time × gender interactions were found for weight (*p* < .001), shape (*p* < .001), and eating concerns (*p* = .002). Only girls saw significant differences between groups over time. No effect post-intervention. Significant group × time × gender interactions were found for body dissatisfaction (*p* < .001, *d* = 0.17). Media Smart (*d* = 0.26) and Life Smart (*d* = 0.33) boys showed a significant improvement post-intervention for body dissatisfaction compared to control group. *Secondary outcomes* Significant group × time × gender interactions were found for depression (*p* = .021), perceived social pressure (*p* < .001), dietary restraint (*p* = .001), and regular eating (*p* = .046). No significant differences post-treatment for boys or girls. Significant group × time × gender interactions were found for perfectionism (*p* = .013). No significant differences post-treatment for girls, but Media Smart boys showed significant improvements in perfectionism post-intervention (*d* = 0.40). Significant interaction effect (group × time × gender) was found for media internalization (*p* = .024). For boys only, Media Smart participants showed significant improvements post-intervention compared to Life Smart (*d* = 0.44) and control group participants (*d* = 0.68). HELPP participants showed significant improvements post-intervention compared to control condition (*d* = 0.47). Significant group × time × gender interactions were found for weight-related peer teasing (*p* = .018). Media Smart boys showed significant reductions in weight-related teasing post-intervention compared to HELPP boys (*d* = 0.57). Life Smart participants reported significantly more daily physical activity than control participants post-intervention.	*Primary outcomes* Both Media Smart and HELPP girls scored significantly lower than Life Smart but not control girls in both weight (MS: *d* = 0.34; HELPP: *d* = 0.43) and shape concern (MS: *d* = 0.33; HELPP: *d* = 0.42) at the 12-month follow-up. For eating concern, both Media Smart (*d* = 0.47) and control girls (*d* = 0.35) scored significantly lower than HELPP girls at 6-months while control girls scored lower than Life Smart girls (*d* = 0.33) at 12 months. No significant differences for boys or girls in body dissatisfaction between groups at either follow-up. *Secondary outcomes* Boys in Media Smart (*d* = 0.51) and HELPP (*d* = 0.46) interventions showed significantly lower depression scores than Life Smart participants but not control group at 6-months follow-up. At 12-months follow-up Media Smart boys scored significantly lower depression scores than the control group (*d* = 0.39). Girls in Media Smart (*d* = 0.45) and control (*d* = 0.44) interventions scored significantly lower perceived social pressure than HELPP girls at 6-months. No significant differences in perfectionism between groups or boys and girls at either follow-up. Boys in Media Smart saw significant improvements in media internalization scores at both 6-month (*d* = 0.61) and 12-months follow-up (*d* = 0.46) compared to control group boys. Media Smart boys saw significant improvement at 6-month follow-up compared to Life Smart boys (*d* = 0.44). HELPP participants showed significant improvements at 6-month follow-up compared to control condition (*d* = 0.50). Media Smart participants reported significantly more exercise than both HELPP and control participants at 6-month follow-up.
Wilksch et al. [117]	**Name of intervention**: Media Smart, Life Smart, and HELPP **Duration**: 8 × 50 min group sessions over 4 weeks **Content**: See Wilksch et al., [116] for content of interventions. **Delivery**: Facilitated by postgraduate psychology students. Overview of the topic, videos, group discussions, presentations (by students), and role plays. **Control**: Class as usual	*Primary outcomes* Significant group × risk × time interactions were found for weight (*p* = .003), shape (*p* = .010), and eating concerns (*p* = .009). Media Smart participants with high SWC scored significantly lower eating concerns than both control (*d* = 0.60) and Life Smart (*d* = 0.58) participants post-intervention. A significant interaction effect (group × risk × time) was found for body dissatisfaction (*p* = .010). Participants in Media Smart (*d* = 0.34) and Life Smart (*d* = 0.31) with low SWC scored significantly lower body dissatisfaction than control group post-treatment. *Secondary outcomes* A significant interaction effect (group × risk × time) was found for depression in boys only (*p* = .043). No significant differences post-treatment. Significant interaction effect (group × risk × time) was found for dietary restraint (*p* = .038) and perfectionism (*p* = .001). Post-hoc testing found no significant differences but lower scores found for lower SWC Media Smart compared to control participants. Significant interaction effect (group × risk × time) was found for regular eating (*p* = .036) and physical activity (*p* = .003). No significant interaction effects for media internalization (*p* = .564), weight-related peer teasing (*p* = .104), or perceived social pressure (*p* = .347).	*Primary outcomes* Significant differences observed in high SWC participants only. Media Smart (*d* = 0.58) and control (*d* = 0.46) participants scored significantly lower eating concerns than HELPP participants at 6-month follow-up. At 12-months follow-up Media Smart (*d* = 0.58) and control (*d* = 0.55) participants scored significantly lower eating concerns than Life Smart participants. Media Smart (*d* = 0.38) and HELPP (*d* = 0.35) participants scored significantly lower than Life Smart participants for weight concerns at 12-months follow-up. Media Smart participants scored significantly lower than Life Smart (*d* = 0.36) participants for shape concerns at 12-months follow-up. No significant differences in body dissatisfaction between low and high SWC or groups at either follow-up. *Secondary outcomes* Media Smart boys with low shape and weight concerns reported significantly lower depressive symptoms at 12-month follow-up than both Life Smart (*d* = 0.26) and control boys (*d* = 0.28). In high SWC participants only, both Media Smart (*d* = 0.40) and control participants (*d* = 0.43) regular eating significantly improved compared to HELPP participants at 12-month follow-up. Control participants significantly improved regular eating compared to Life Smart participants at 12-month follow-up (*d* = 0.34). At 6-month follow-up Media Smart participants reported significantly more physical activity than control (*d* = 0.38) and HELPP participants (*d* = 0.54).
Wiseman et al. [118]	**Duration**: 5 x group sessions over 6 weeks. Length of sessions unknown. **Content**: *Session 1*: healthy living and issues other than ***body weight***,*** shapes***,*** and dieting***. Discussion around media figures that are healthy and unhealthy. Importance of ***exercise***, hygiene, ***nutrition***, sleep, and ***mental health*** in maintaining a healthy body. *Session 2*: **Social media**, magazine photographs of female models, and importance of valuing oneself. *Session 3*: nutritional guidelines and challenging perceptions of their sleep, dietary, and ***physical fitness*** needs. The topic of dieting, its popularity and misconceptions were discussed. *Session 4*: what are EDs and their physical and mental consequences. *Session 5*: what is a healthy body, eating healthily and healthy exercise. Valuing oneself other than by appearance and what are the consequences of dieting? **Delivery**: Facilitated by psychologist/psychiatrist. Overview of the topic, group discussions, critical reflection, and in-class activities. **Control**: Health lectures	*Primary outcomes* Drive for thinness changed significantly over time in both conditions (*p* < .001). Italy participants in the intervention group showed a significant decrease in drive for thinness (*p* = .017) at post-intervention compared to the control condition. No other significant effects were found for Italy or USA participants. *Secondary outcomes* No significant effects of intervention, country, time or any interactions for self-esteem or eating behaviors.	No follow-up.

AMC: Aesthetic Model Criticism; BMI: Body Mass Index; CIMEC: Questionnaire on Influences of Aesthetic Body Ideal; DT: drive for thinness; EAT: Eating Attitude Test; ED: eating disorder; EDE-Q: Eating Disorders Examination Questionnaire; EDI: Eating Disorder Inventory; ES: effect size; HELPP: Helping, Encouraging, Listening and Protecting Peers Initiative; HRHM: higher-risk and higher-motivated group; HRHM-C: higher-risk and higher-motivated combined group; ICC: intra-cluster correlation; ML: media literacy; NUT: nutrition; Other-C: Other-combined group; PIA-2: prevenci onintegrada de trastornos alimentario y obesidad [Integrated prevention of eating disorders and obesity]; SWC: Shape and weight concerns; WLC: waitlist control

^a^Authors combined Classes 1 and 3 to form a single HRHM-only group (HRHM-C) and a single Other-only (Other-C) group. HRHM-C and Other-C are analyzed separately without statistical comparison between them

^b^Authors did not report which effect size measure was used. Authors were emailed but reviewers did not receive a reply

^c^Authors combined the co-educational and single-sex intervention conditions to create one intervention condition to compare against the non-treatment control

^d^Adjusted for clustering (ICC applied but not reported)

^e^Analysis uses change scores

^f^Adjusted for clustering (ICC applied and reported)

### Eating disorder psychopathology outcomes

Eating Disorder psychopathology was assessed using validated tools including EDE-Q [24], children’s version of the Eating Disorder Examination Questionnaire [48], Eating Attitudes Test [36], Eating Disorder Inventory [33, 34, 35], and Eating Disorder Diagnostic Scale [102]. Five studies employed more than one of these instruments [1, 20, 112, 116, 117]. All interventions included content addressing weight, shape, or eating concerns.

For global ED psychopathology outcomes, measured using the EDE-Q and EAT, two studies reported small between-group improvements post-intervention [37, 87]; *d* = 0.32) and one at 7-month and 30-month follow-up [37]. Within-group improvements were observed post-intervention in one study [20]. Among the remaining studies that reported global ED psychopathology outcomes, none demonstrated significant improvements [42, 89, 104, 112]. Significant improvements were observed in interventions that included content on social media, eating, weight, and shape concerns, historical beauty standards, and nutrition.

Most studies utilized subscales of validated tools to measure ED psychopathology. Across studies, subscale reporting was inconsistent, with some trials demonstrating improvements while others found no significant changes. Post-intervention between-group improvements were observed across subscales of body dissatisfaction [22, 114, 116]; drive for thinness [114, 118]; bulimic symptoms [28]; and oral control [87], with effect sizes ranging from small *d* = 0.33 to moderate (*η*^2^ = 0.06). Shape, weight, and eating concerns significantly improved between-groups in one study post-intervention and at follow-up, with effect sizes ranging from small (*d* = 0.31) to moderate (*d* = 0.60) [117]. Within-group improvements were also observed post-intervention in drive for thinness and shape and restraint concern [1]. Eight studies demonstrated no significant improvements across several subscales [1, 62, 89, 93, 96, 112, 114, 118].

In studies comparing girls and boys, on average, girls showed greater improvements than boys. Girls showed post-intervention improvements in ED psychopathology across five studies [20, 42, 104, 112, 116]. Where reported, effect sizes indicated small effects ([104]: *d* = 0.21; [112]: *d* = 0.23). However, one study found increased ED psychopathology in 8th -grade girls [23]. Two studies observed small improvements in boys’ outcomes post-intervention [42, 116], with effect sizes ranging from *d* = 0.26 and *d* = 0.33 [116].

### Physical health outcomes

Physical health outcomes only included BMI. Three studies measured changes finding no significant differences across conditions and time. Two studies included girls only [28, 62] whereas Pérez-Vázquez et al. [87] included a mixed-gender sample. One study recorded follow-up data, but no significant differences were reported [62]. BMI was measured by researchers in two studies [62, 87]; however, one study did not specify how BMI was obtained [28].

### Broader psychological well-being outcomes

Studies utilized several broader psychological outcomes including weight management behaviors, body image, and psychological distress.

#### Weight management behaviors

**Eating behaviors**. Five trials measured eating behaviors using multiple questionnaires, but only two incorporated eating behavior content within the intervention [114, 118]. Between-group improvements were observed in three studies. High-risk or high SWC participants reported significant improvements in regular eating at follow-up in both the Media Smart intervention (*d* = 0.40) and associated control groups (*d* = 0.43, *d* = 0.34) [114, 116, 117]. No change was noted in purging behaviors [114]. No gender × SWC interaction analyses were reported for dietary restraint or regular eating [116, 117]. Of the remaining two studies, neither demonstrated significant improvements [22, 118].

**Exercise**. Three studies assessed exercise, with all three interventions including content related to physical activity. One study reported minimal between-group improvements in the emotional element of exercise (how physical activity affects mood, emotions, and psychological well-being) post-intervention (*d* = 0.17) and at 12-month follow-up (*d* = 0.16) [112]. The amount of physical activity significantly increased for adolescents accessing the Media Smart intervention compared to the control (d = 0.38) and HELPP condition (d = 0.54) at 6-month follow-up [116, 117].

**Nutrition**. Two studies measured nutritional outcomes [89, 104]. Nutrition outcomes included knowledge of nutrition and frequency of protein and creatine supplement usage and diet aid use. Nutritional knowledge showed moderate improvements between-groups (*g* = 0.60), but not maintained at follow-up [89]. Frequency of protein and creatine supplement usage remained stable in active condition but increased at both follow-up timepoints in control condition [104]. No effects were observed for diet aid use. Nutritional content was included in both interventions.

#### Body image and acceptance

**Beliefs about one’s own body**. Five trials measured adolescents’ views on their own body image. All five interventions included content addressing body image. Improvements were observed in one study across body image flexibility at post-intervention (girls; *d* = 0.18), 3-month (boys; *d* = 0.34) and 12-month follow-ups (girls; *d* = 0.47) [104]. Between-group improvements in body esteem were observed at follow-up, but not post-intervention [96]. Between-group improvements in internalization of the thin-ideal were observed post-intervention in three studies: a large effect was reported by Svantorp-Tveiten et al. ([104]; *η*^2^ = 0.26), a small effect by Dunstan et al. ([22]; d = 0.17), and additional improvements were observed in Sharpe et al. [96]. These effects were sustained at follow-up across all three studies [22, 96, 104]. Although improvements were noticed across girls and boys, significant effect remained for girls at 3-month (*d* = 0.40) and 12-month follow-ups (*d* = 0.44) [104]. Separately from the gender comparisons, the same study reported minimal between-group improvements in drive for leanness (*d* = 0.12) and muscular internalization post-intervention (*d* = 0.15) [104]. One study reported within-group improvements [93] and another study found no significant improvements in internalization of the thin-ideal [42].

**Perceived pressure on body image**. Eight trials assessed adolescents’ body image comparisons, appearance-related talk, and perceived sociocultural pressure. All eight interventions included content addressing body image. Two studies reported small between-group improvements in appearance comparison post-intervention [22, 112] and one study at follow-up [22]. Four studies reported outcomes on perceived pressure from the media, with between-group improvements consistent across three studies [104, 112, 116]. However, findings across studies indicate notable gender disparities, with effect sizes ranging from small *d* = 0.44 to moderate *d* = 0.68 [116]. Four studies assessed perceived sociocultural pressures on body image, with mixed findings. Two studies reported between-group improvements [37, 116]; whilst two studies found no significant improvements on this measure [42, 117]. Lastly, two studies found no significant improvements on body image conversations [22, 96].

**Body shaming/weight related teasing**. Four trials measured weight-related teasing. All four interventions included content on addressing body shaming or weight-related teasing. Only boys in the Media Smart intervention observed moderate between-group improvements post-intervention (*d* = 0.57) [116]. Of the remaining studies none demonstrated significant improvements [22, 112, 117].

#### Psychological distress

**Mood**,** depression**,** and anxiety**. Five studies assessed psychological distress, with mixed findings. Three interventions included content on emotions [42, 116, 117]. Between-group improvements in anxiety were observed post-intervention in one study (*d* = 0.11), but not maintained at follow-up [42], whereas anxiety and depression improved at 12-month follow-up in another study (*d* = 0.31), but not post-intervention [104].

The Media Smart intervention consistently improved outcomes for boys’ depressive symptoms than comparison conditions at both 6-month follow-up (*d* = 0.51) and 12-month follow-up (*d* = 0.39) [116]. Boys with low SWC in the Media Smart intervention yielded improvements in depressive symptoms at 12-month follow-up (*d* = 0.26) [117]. Between-group improvements were also observed in the HELPP intervention (*d* = 0.51) at 6-month follow-up [116]. One study found no significant improvements in psychological distress [96].

**Self-esteem and self-identity**. Seven trials measured self-esteem and self-identity, with five interventions including content on self-esteem [20, 42, 87, 104, 114]. Two interventions showed small improvements between-groups post-intervention (*η*^2^ = 0.04; *d* = 0.15) [22, 104] and at 12-month follow-up (*d* = 0.33) [104]. Within-group improvements were observed in one study [20]. Among the remaining studies that reported post-intervention self-esteem outcomes, none demonstrated significant improvements [42, 87, 114, 118].

**Perfectionism**. Perfectionism was measured in two studies, but none of the interventions included content on perfectionism. Whilst significant improvements (*d* = 0.40) in perfectionism were observed post-intervention for boys in the Media Smart intervention, this was not maintained at follow-up [116]. Wilksch et al. [117] reported SWC was a moderator between group and time, but the breakdown of this effect was not reported.

**Social support**. Only one study assessed social support but found no significant differences in perceived support from friends between the intervention or control group overall [96].

## Discussion

This systematic review evaluated the effectiveness of universal secondary school-based interventions addressing transdiagnostic core ED psychopathology and at least one other psychological risk factor. Overall, most universal school-based ED interventions produced some immediate improvement in ED symptoms or well-being but effects were generally small or rarely sustained at follow-up. Importantly, the high risk of bias in most studies, as assessed by the RoB-2 tool [99] indicates methodological weaknesses which limit the confidence of findings. Thus, results from this review should be interpreted with caution.

Interventions were more effective at improving body dissatisfaction and weight, shape, or eating concerns compared to other ED symptomatology or broader well-being outcomes, likely reflecting their emphasis on body image, dieting, and self-esteem. Among the interventions reviewed, Media Smart [116, 117] showed comparatively stronger between-group effects. This may reflect its explicit focus on how media shapes self-image and its practical, skills-based strategies, an important component of programs for CYP [65]. It also emphasizes recognizing and managing harmful appearance-related content, a focus that has become increasingly relevant given the substantial rise in adolescents’ social media use, particularly since COVID-19 [64, 91]. Media literacy approaches have been found to be effective ED universal prevention interventions [56] and have long been advocated as a mechanism for reducing body dissatisfaction and disordered eating by promoting critical evaluation of media messages and reducing thin-ideal internalization [53, 93, 115]. However, because [116, 117] analysed the same dataset in both publications, the increased risk of false positives associated with repeated analyses should be acknowledged. Nonetheless, given the substantial rise of appearance-focused platforms such as Instagram, TikTok, and Snapchat [107], researchers and schools may benefit from incorporating media literacy that addresses both self-image and strategies for managing online content, and by expanding their focus to digital image-based and algorithm-driven environments [39].

Facilitators and program duration varied substantially across trials, and we found no consistent pattern linking either factor to intervention effectiveness, a result that mirrors previous reviews reporting similarly inconsistent associations between delivery format and outcomes [63]. However, feedback from CYP indicate a clear preference for interventions delivered by trained professionals with mental-health expertise and suggest sports or health teachers may be appropriate facilitators when adequately trained, given their existing rapport with students [66]. Additionally, CYP indicate value in longer or additional sessions [65]. Given the limited time in school curriculum and other competing demands [94], finding from this review and previous research offer teachers flexibility in the implementation of interventions.

The interventions included in this review incorporated a wide range of components, including facilitated discussions and critical analysis, practical in-class activities (e.g., watching videos, letter or journal writing, and roleplays), and homework tasks. Feedback from CYP emphasize the value of opportunities for discussion and reflection within sessions and suggest programs could be strengthened by increasing interactive elements, offering sensory tools to support engagement, and providing additional incentives [65]. Future research would therefore benefit from drawing on the components used in existing interventions while more systematically incorporating the preferences of CYP in program design.

Although most interventions produced some immediate improvement in ED symptoms or well-being, both between-groups and within-groups, effects were generally small or rarely sustained at follow-up. There were no significant changes in BMI. These findings are consistent with previous reviews [11, 95, 121] which similarly reported small effect sizes, low-quality evidence, and only modest improvements in ED symptoms and related outcomes. The mixed and often small effects observed across ED universal prevention trials raise important questions about the underlying mechanisms of change.

One possibility is that gender-specific and cultural differences are often insufficiently addressed, potentially limiting the relevance and effectiveness of program content for diverse groups. Consistent with previous reviews [17, 88], the current review found greater benefits for girls than boys, suggesting that the absence of gender-tailored content may partly account for mixed or null effects [81]. Although core risk factors overlap, differences in clinical presentation, girls’ pursuit of thinness versus boys’ drive for muscularity, may explain disparities observed across studies [29, 54, 76]. Male-specific content targeting the ‘male appearance ideal’ and themes such as steroids have yielded medium to large effects [51], demonstrating the value of tailored approaches. Yet gender is not binary, and gender-affirming approaches for transgender and gender-diverse young CYP, ideally delivered by clinically trained facilitators, are warranted [38, 50].

Culturally, samples in this review were predominantly Caucasian. Exclusion of non-English studies likely exacerbated the underrepresentation of diverse contexts, and where other ethnicites were included, they comprised small subsamples. Despite limited research in non-Western settings [5], EDs are prevalent and whilst individuals experience weight, shape, or eating concerns, culture-specific factors are important factors to consider [4, 71, 77]. These differences constrain external validity and call for intersectional, culturally responsive delivery by competent clinicians and flexible models that address contextual factors such as stigma and relational dynamics [2, 15]. In sum, the mixed and often small effects likely reflect inadequate gender tailoring and cultural fit. Future universal interventions should be explicitly gender-responsive and culturally competent to improve impact and effectiveness.

Secondly, the broad scope of universal interventions and ambiguity surrounding their ultimate aims may limit their capacity to produce meaningful change. The mere identification of specific ED risk factors does not imply uniform pathways to difficulties. Such heterogeneity in how these risks operate across individuals likely contributes to the mixed findings observed in this and previous reviews [40, 63]. A modular approach to prevention, allowing CYP to select content aligned with their needs or interests (e.g., body image, social media, sleep, general well-being) while providing evidence-based mechanism-focused modules for those wanting, or requiring more targeted support, may offer a more responsive alternative to purely universal provision [18, 40]. Evidence indicates that when multitiered delivery systems are implemented, they can reduce problem behaviors and enhance student engagement and academic outcomes [59]. Adopting this approach may also facilitate testing of specific theoretical pathways which will continue to enhance understanding of the most effective factors for reducing ED symptomatology in universal interventions.

Given the broad scope of universal interventions, identifying outcome measures that meaningfully capture population-level effects and align with active mechanisms is crucial [40, 63]. Although ED universal programs target proximal constructs such as body image, media literacy, or self-esteem, trials often assess ED symptomatology as a primary outcome, despite floor effects that limit the detectability of meaningful change in universal samples [6, 56]. Consequently, the small effects observed in this, and previous reviews may reflect not only limitations in intervention design but also misalignment between theoretical targets and outcome measures, as well as short follow-up periods [63]. For example, the lack of effects for BMI in this review likely reflects participants’ BMI scoring within the ‘healthy range’, suggesting substantial changes were neither necessary nor expected. Short follow-up periods in population-level trials limit the ability to detect meaningful or sustained impacts [40]. Evaluating proximal changes and examining how these may (or may not) translate into symptom reductions over time could provide a clearer picture of what universal interventions can realistically achieve [63]. Additionally, longer follow-ups and longitudinal research that track CYP across their school years are warranted to understand the durability and developmental relevance of intervention effects [40, 63]. Researchers could also consider parental reports given CYP can under-report symptoms [52] or utilize male specific outcomes considering measures were developed and validated in women [75].

Finally, the possibility that some universal school-based interventions may have limited benefit, or in certain subgroups, unintended negative effects, warrants careful consideration. In one review, around one-third of high-quality trials reported at least one negative outcome, suggesting that adverse effects are not rare outliers but a pattern within universal approaches [43]. In this review many of the interventions primarily addressed negative aspects of body image and a couple of studies reported worsening effects in ED symptomatology [42, 62]. Whilst these approaches aim to raise awareness, they may inadvertently highten attention to appearance or reinforce preoccupation with it, thereby sustaining the very cultural norms they seek to challenge [43]. Considering the protective influence that positive body image constructs can have against ED symptoms [60], future research may warrant focusing on positive body image components rather than negative aspects of body image, or including trigger and content warnings [66]. However, although harms have been reported in previous research, these are not attributed to inherently harmful content, but to the processes or contexts through which some CYP engage with the material [30].

Measurements used to report changes may have unintended negative effects. In this review, physical health outcomes were measured by BMI. However, the s.p utilization of BMI in CYP, particularly those without an ED, has commonly cited criticism [105]. Firstly, atypical EDs are the most common subtype [79], nor does one’s body size predict the development of an ED [80]. Whilst weight and BMI can be useful in clinical settings [106], concerns regarding weight-stigma and possible development of disordered eating have been raised in school-based programs [106, 110]. Universal interventions therefore may inadvertently amplify symptom awareness, encourage rumination, or trigger unhelpful social comparison, creating pathways for iatrogenic effects rather than prevention [7, 31, 43, 109].

Taken together, the above issues highlight the need for more nuanced, tailored, and theoretically grounded approaches in future ED universal prevention research. In line with recent recommendations for universal interventions [18, 30, 63], we suggest the following priorities for future research and practice: (1) embed gender-diverse and culturally specific content (noting heterogeneous responses and underrepresentation of minoritized groups); (2) centre the voice of CYP in co-design to improve acceptability and avoid iatrogenic pathways; (3) use validated outcome measures that are sensitive to mechanisms of change in non-clinical settings; and (4) consider a modular, needs-led universal offer (e.g., CYP choose targeted body image/ED modules or neutral well-being content such as sleep/physical activity), reserving more intensive elements for those likely to benefit and explicitly testing for differential effects.

## Strengths and limitations

This review has several important strengths including a comprehensive search strategy and inclusion of gold standard RCT methodology [47]. Seven studies were also assessed in previous reviews [11, 17]. Overall bias ratings are consistent with those reported earlier, suggesting similar methodological limitations identified across independent researchers. This review builds on previous syntheses [11, 17, 121] by expanding the focus to universal interventions targeting ED symptomatology in addition to other psychological risk factors.

Despite the strengths, this review is not without limitations. Firstly, in line with systematic review recommendations [111], future reviews should include non-English and gray literature studies. The absence of meta-analytic techniques limits synthesis to a more narrative and subjective interpretation of findings without the combination of s.p synthesizing results with statistical models [16]. This review also did not conduct sensitivity analyses, which limits the ability to assess the robustness of findings or account for potential clustering-related biases, such as those introduced by intra-cluster correlation (ICC). Additionally, although researchers were transparent and reported deviations of intended methods, the refinement of inclusion criteria limits the breadth of available evidence and the generalisability of findings.

There were also limitations with the sample papers. Across the included trials, clusters were defined at the level of classroom (*n* = 8) or school (*n* = 9), each carrying different implications for the degree of similarity among participants. These clustering structures may influence outcomes because students within the same class or school are not independent. Only two studies adjusted for clustering effects in their analysis [42, 93] and only Rutsztein et al. [93] reported ICC’s. The lack of consistent adjustment and limited ICC reporting reduce confidence in the precision of effect estimates and may inflate statistical significance. Most studies did not report SES and ethnicity data and given the continued under-representation of non-western cultures in literature, future research should accommodate further subanalyses of minoritised groups. The RoB-2 tool highlighted methodological concerns, particularly that the process of randomization remains problematic and high-risk [68]. However, conducting high-quality RCTs in schools presents unique challenges [103], with informed consent and non-blinding of facilitators inevitably increasing bias. To improve write-up of the randomization process, researchers suggest 100–200 protected words to allow for adequate description of randomization [68].

Only seven papers had a study design directly comparing the intervention to an active control, with two of these seven by Wilksch et al. [116, 117]. While WLC and CAU conditions are acceptable in the early stages of trials, they can compromise the quality of outcome data [84]. Robust active comparisons are essential to determine how universal ED prevention interventions can most effectively support schools. Additionally, the description of interventions also lacked clarity at times, making it difficult to establish the inclusion of core ED psychopathology. Many studies referred to the term ‘body image’ but it remained unclear whether the focus was on weight, shape, or eating concerns or a broader emphasis around appearance-related concerns [13, 32, 57]. Authors therefore adopted a conservative approach and studies that clearly stated the inclusion of core ED psychopathology were included. Thus studies might have unknowingly been excluded on this basis. Some studies too selectively reported the analysis undertaken; whilst this occurred in older papers and on secondary aim outcomes, this reporting bias has the potential to skew conclusions [55]. Taken altogether, future studies should consult CONSULT guidelines for reporting RCTs to ensure the integrity of evidence-based research [49].

## Conclusions

Overall, most universal school-based ED interventions produced some immediate improvement in ED symptoms or well-being but effects were generally small or rarely sustained at follow-up. No significant changes in BMI were observed, although participants were generally of healthy weight at baseline. Girls tended to benefit more than boys despite the absence of gender-specific content in the included interventions. However, the high risk of bias limits the confidence of findings. Future research would benefit from adopting active comparison groups and longer follow-ups. Whilst core ED psychopathology in addition to the risk factors of body image dissatisfaction, dieting, self-esteem, and social media appear consistent factors to include in universal interventions, future researchers may wish to adopt a modular approach to facilitate testing of specific theoretical pathways and to enhance our understanding of the most effective factors in universal school-based ED interventions to ultimately reduce the incidence of EDs in society.

## Data Availability

Data sharing is not applicable to this article as no new data were created or analyzed in this study.

## Abbreviations

AMC: Aesthetic model criticism
BMI: Body mass index
CYP: Children and young people
CAU: Class as usual
RoB-2: Cochrane risk of bias
EAT: Eating attitudes test
ED: Eating disorder
EDDS: Eating disorder diagnostic scale
EDE-Q: Eating disorder examination questionnaire
EDI: Eating disorder inventory
HELPP: Helping, encouraging, listening and protecting peers initiative
HRHM-C: Higher-risk and higher-motivated combined group
ICC: Intra-cluster correlation
ML: Media literacy
ML + NUT: Media literacy + nutrition
MH: Mental health
PRISMA: Preferred reporting items for systematic reviews and meta-analyses
RCTs: Randomised controlled trials
SWC: Shape and weight concern
SES: Socioeconomic status
WLC: Waitlist control
WHO: World Health Organization
